# Dynamics of necklace beams in nonlinear colloidal suspensions

**DOI:** 10.1038/s41598-017-12169-x

**Published:** 2017-09-15

**Authors:** Wiktor Walasik, Salih Z. Silahli, Natalia M. Litchinitser

**Affiliations:** Department of Electrical Engineering, University at Buffalo, The State University of New York, Buffalo, New York, 14260 USA

## Abstract

Recently, we have predicted that the modulation instability of optical vortex solitons propagating in nonlinear colloidal suspensions with exponential saturable nonlinearity leads to formation of necklace beams (NBs). Here, we investigate the dynamics of NB formation and propagation, and demonstrate a variety of optical beam structures emerging upon vortex beam propagation in engineered nonlinear colloidal medium. In particular, we show that the distance at which the NB is formed depends on the input power of the vortex beam. Moreover, we show that the NB trajectories are not necessarily tangent to the initial vortex ring, and that their velocities have components stemming both from the beam diffraction and from the beam orbital angular momentum. We also demonstrate the generation of elliptical rotating solitons and analyze the influence of losses on their propagation. Finally, we investigate the conservation of the orbital angular momentum in necklace and elliptical rotating beams. Our studies, performed in ideal lossless media and in realistic colloidal suspensions with losses, provide a detailed description of NB dynamics, and may be useful in analysis of light propagation in highly scattering colloids and biological samples.

## Introduction

Structured light, especially optical vortices carrying orbital angular momentum (OAM)^1^, have attracted a growing attention in the last two decades. A particular interest was paid to generation of spiraling light patterns naturally stemming from the twisted nature of the OAM beams. In the linear regime, spiraling beams can be created by the interference of two vortices with opposite charges and different longitudinal propagation constants^2–7^. In nonlocal nonlinear media, stable spiraling solitons were found. They can originate from optical fields carrying a nonzero OAM^8–14^, or from a collision of skewed solitons that do not carry OAM^15–19^. A broad review of vortex solitons and their interactions can be found in refs^20,21^.

Another type of beams that originate from optical vortices are necklace beams (NBs)—beams with azimuthal modulation in their intensity profiles. Such beams can be created by interference of optical vortices with different charges^22–25^, or can be a result of a modulation instability induced (MI-induced) splitting of a vortex^26–30^, or a super-Gaussian beam^31^. Solitons building a NB have been shown to either escape away from the center of the NB, following trajectories tangent to the NB ring^26,27^, or to fuse and create a vortex pattern^32^, depending on their arrangement and phase distribution.

Colloidal suspensions (CSs) constitute a useful platform to study nonlinear light-matter interactions. Many interesting effects in CSs have been reported, such as optical trapping of dielectric particles^33–37^ that found applications in optical tweezing, and creation of artificial nonlinear media^38,39^. The initial prediction of the Kerr-like nature of the nonlinearity of CSs was later refined. It was shown that the nonlinearity of CSs has exponential character and can be either supercritical or saturable^40,41^. The nonlinearity is supercritical for particles with positive polarizability (refractive index of a particle *n*_*p*_ higher than the background index *n*_*b*_). The high-index particles are attracted to the high-intensity region leading to local increase of the effective refractive index. The nonlinearity is saturable for negative polarizability particles (*n*_*p*_ < *n*_*b*_). In this case, the low-index particles are repelled from the high-intensity region increasing the local effective index and resulting in self-focusing nonlinearity. Propagation, stability, and interactions of Gaussian beams^42–47^ and optical vortices^30,48,49^ in dielectric and metallic nonlinear CSs have been extensively studied both theoretically and experimentally.

In our previous work, we have investigated the propagation of vortex beams in nonlinear CSs, and have shown that NBs can be generated via the MI^30^. There, the initial vortex beam corresponded to a stable solution of a nonlinear Schrödinger equation defined by a fixed vortex radius, and associated charge and power level. In this Letter, we demonstrate the generation of NBs from vortices that are not stationary solutions. This approach offers more freedom in the choice of the input beam parameters, and allows us to modify the vortex power, while keeping the charge and the radius fixed. In the first part, we show how the distance at which the instability onsets depends on the initial vortex power. Moreover, we study the trajectories of the solitons forming the NB and analyze the evolution of the NB profile for fixed power, depending on the charge of the vortex and its radius. We demonstrate the generation of elliptical rotating solitons resulting from the fusion of two solitons resulting from an interference of two vortices. Finally, we investigate the OAM conservation and the role played by losses in all the phenomena described above.

We study the propagation of beams carrying the OAM in a CS built of air bubbles with refractive index *n*_*p*_ = 1 uniformly distributed in water with the refractive index *n*_*b*_ = 1.33, resulting in a negative polarizability of the bubble. We note that in the laboratory experiments, one can use polytetrafluoroethylene (PTFE) particles dispersed in a glycerin–water solution to realize a stable negative polarizability CS. The volumetric filing fraction of this solution is *f* = 1‰ and the radius of the air bubbles is taken as *r*_*p*_ = 50 nm. The suspension is assumed to be at the room temperature *T* = 293 K and the incoming free-space wavelength of light is *λ*_0_ = 2*π*/*k*_0_ = 532 nm. The nonlinearity in such a system has exponentially saturable character^40^ and the propagation of the slowly varying envelope of the electric field *ϕ* can be described by:

1 $$i\frac{\partial \varphi }{\partial z}+\frac{{\nabla }_{\perp }^{2}\varphi }{2{k}_{0}{n}_{b}}+[{k}_{0}({n}_{p}-{n}_{b}){V}_{p}+\frac{i}{2}\sigma ]{\rho }_{0}{e}^{\frac{\alpha }{4{k}_{B}T}|\varphi {|}^{2}}\varphi =\mathrm{0,}$$

where *z* denotes the propagation distance, ${\nabla }_{\perp }^{2}$ is the transverse Laplacian, ${V}_{p}=4\pi {r}_{p}^{3}\mathrm{/3}$ is the volume of the particle, and *k*_*B*_ is the Boltzmann constant. The scattering cross section responsible for loss in the Rayleigh regime is given by $\sigma =128{\pi }^{5}{r}_{p}^{6}{n}_{b}^{4}{({\gamma }^{2}-\mathrm{1)}}^{2}\mathrm{/[3}{\lambda }^{4}{({\gamma }^{2}+\mathrm{2)}}^{2}]$ ^40^, where *γ *= *n*_*p*_/*n*_*b*_. The unperturbed particle concentration is ${\rho }_{0}=f/{V}_{p}$, and the particle polarizability is $\alpha =3{V}_{p}{\varepsilon }_{0}{n}_{b}^{2}({\gamma }^{2}-\mathrm{1)/(}{\gamma }^{2}+\mathrm{2)}$, where *ε*_0_ denotes the vacuum permittivity.

**Figure 1:**
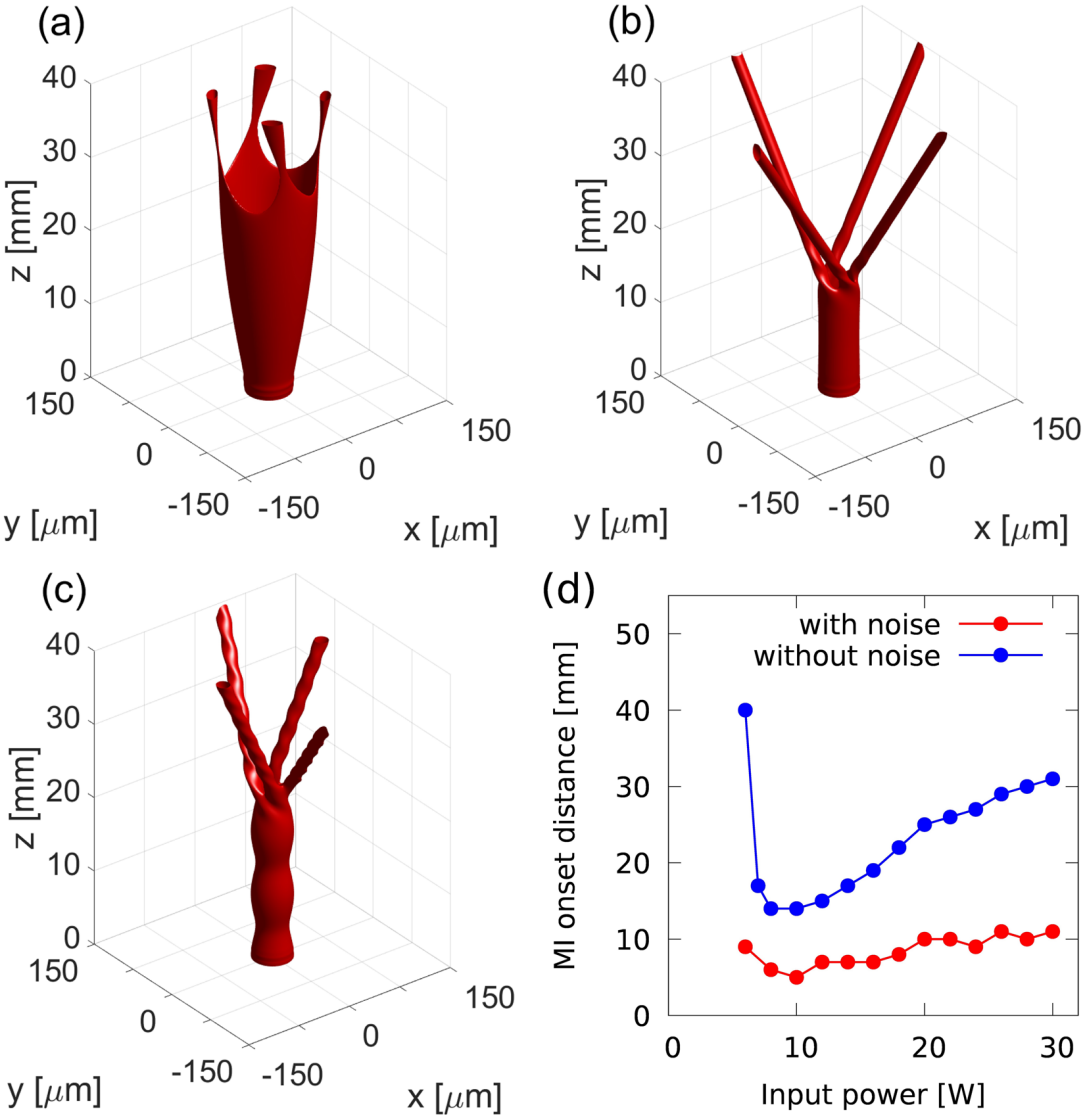
(**a–c**) Iso-intensity surfaces for a charge-two vortex beam with the initial radius *R* = 20 *μ*m and powers 6 W (**a**), 8 W (stable vortex) (**b**), and 18 W (**c**), for the input without the noise. Propagation losses are neglected (*σ* = 0). (**d**) Dependence of the distance at which the MI onsets as a function of the power of the vortex for input beams with 5% of white noise (red) and without noise (blue).

First, we study the dependence of the distance at which the MI onsets as a function of the power of the vortex with the profile of a soliton originating from the Laguerre–Gaussian profile with a fixed charge *m* = 2 and radius *R* = 20 *μ*m (measured from the center of the vortex to the location of the peak intensity). Typical iso-intensity surfaces obtained during a 40-mm-long propagation are shown in Fig. 1(a)–(c). For all the iso-intensity plots in this Letter, the iso-intensity surfaces are drawn at the value of ${I}_{{\rm{i}}{\rm{s}}{\rm{o}}}={\rm{m}}{\rm{a}}{\rm{x}}\{{\rm{I}}({\rm{x}},{\rm{y}},{\rm{z}})\}/8$. Figure 1(b) shows the case where the input beam is a stable vortex solution. In the first 15 mm of propagation, we can see a stationary propagation of the vortex, during which the transverse profile does not change. After that, the MI onsets due to the presence of the numerical (discretization) noise, and four solitons are generated which travel away from the vortex ring. At the power levels lower than that for the stable solution (<8 W), the diffraction of the vortex beam dominates. As it can be seen in Fig. 1(a), for the power level of 6 W, in the first stage of the propagation, the vortex beam diffracts but the MI still manages to create the NB in a later stage of the propagation. Below this value of power, the MI is not observed and the vortex experiences only linear diffraction.

At the power levels higher than 8 W, the MI onsets at a longer distance than for the stable vortex soliton because of the saturation of the nonlinear effects in the CS. For a perturbation with a given modulation amplitude, the MI-mediated growth is slower in the regime close to the saturation than below the saturation regime, due to different slopes of the intensity dependent refractive index curve, *n*(*I*), in these two regimes (for more details, see the Supplementary Materials). Figure 1(c) illustrates the evolution of the vortex beam at a high-power level, close to the nonlinearity saturation. At first, due to the excess of power, the radius of the vortex soliton oscillates, which resembles the behavior of higher-order solitons. Then, the MI creates a NB built of four separate solitons. However, due to the excess of power in each of the solitons and the presence of the OAM in the beam, the created solitons have an internal twisted structure, that reflects the presence of two closely spaced solitons rotating around a common center of mass.

The plot in Fig. 1(d) summarizes the results of the study of the distance at which the MI onsets in function of the input power. The studies in which no noise was added to the input beam show a clear dependence of the MI-onset distance on the input power. The MI onsets at the shortest distance for the power corresponding to the stable vortex solution. Deviation from this power in any direction results in increase of the MI-onset distance either due to competition with diffraction (lower powers) or saturation effects (higher powers). Addition of a white noise with amplitude of 5% to the input beam accelerates the MI-mediated formation of NBs and leads to a weaker dependence of the MI-onset distance on the input power. Nonetheless, the character of this dependence is the same as in the noiseless case, as it can be seen in Fig. 1(d).

**Figure 2:**
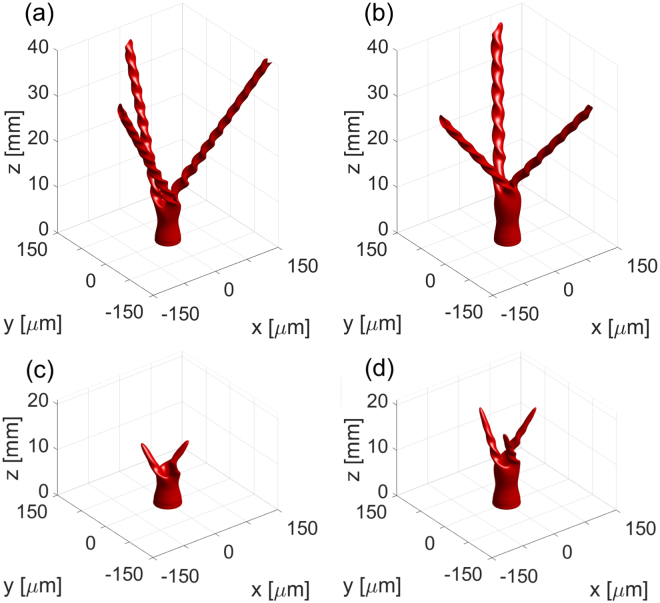
Iso-intensity surfaces for a charge-two vortex beams with radius *R* = 20 *μ*m (same as is Fig. 1) for input powers 20 W (**a**,**c**) and 30 W (**b**,**d**) for the case with 5% of noise added to the input. Panels (a) and (b) show the propagation in the medium without losses, while panels (c) and (d) show the result of simulations where losses are included.

The presence of losses in the system prevents the NB formation at low-power levels but does not dramatically change the MI-onset distance for high-power levels. In Fig. 2, we present a comparison of iso-intensity surfaces for the high-power beams propagating in systems with and without loss. Loss strongly limits the propagation distance of the solitons created via the MI. Increase of the input power results in increased propagation distance of the created solitons due to the nonlinear self-induced transparency in the CS studied here^43^. With the increase of power, the loss experienced by the beam decreases, as the scattering particles with negative polarizability are expelled from the high-intensity region.

The number of beams into which the vortex splits due to the MI is not affected by the input power, for moderate levels of power, as it can be seen in Fig. 1(a)–(c). For powers of 20 W and 30 W, corresponding to more than 2.5 times the power of the stable soliton, the number of beams is reduced to three, and these beams have a strongly twisted character, as illustrated in Fig. 2(a) and (b).

Next, we investigate the trajectories and escape velocities of the solitons forming the NBs. Theoretical models have predicted that the escape velocity of a soliton ${v}_{\perp }$, defined as the distance traveled in the transverse plane (*x*–*y*) divided by the distance traveled along the propagation axis (*z*), is proportional to the absolute value of the charge of the vortex and inversely proportional to the input vortex radius: ${v}_{\perp }\propto |m|/R$ ^23,27^. This prediction is valid for vortex beams with radius *R* much larger than the width of the vortex donut [defined by the parameter *ξ* in eq. 2]^23^. Additionally, up to now, the trajectories have always been reported to be tangent to the NB ring^26,27^. Here, we analyze the validity of this analytical prediction for various radii of the vortex beams, and show that the trajectories can form an arbitrary angle with the line tangent to the NB ring.

Depending on the radius, the power level, and the charge of a vortex, the MI leads to formation of NBs with a different number of maxima^27–30^. Here, we analyze beams where each of the beam parameters can be controlled independently. To achieve that, instead of analyzing the MI-mediated breakup of the input vortex, we start from a NB with a predetermined number of maxima and fixed charge, radius, and input power. Such NBs can be generated using two methods (i) interference of two vortices with different charges but the same radii^22–24^ resulting in a continuous distribution of phase in the azimuthal direction or (ii) arrangement of *N* solitons into a circular pattern where the initial phase of the *j*th soliton is given by 2*πmj*/*N*. Here, *m* denotes the NB charge and *N* is the number of solitons building the NB. This method results in a step-wise distribution of the phase in the azimuthal direction. Despite the fact that the latter method of NB generation creates more stable beams^50–53^, it is more cumbersome from the experimental perspective, as it requires generation of *N* independent solitons with appropriate phases. Therefore, here we use the former technique, which requires only two independent beams regardless of the number of beams building the NB. The input profile with *N* = 8 maxima and charge *m* is described in cylindrical coordinates (*r*, *θ*, *z*) by:

2 $$\varphi (r,\theta ,z=\mathrm{0)}=f(r)[{e}^{i(m+\frac{N}{2})\theta }+{e}^{i(m-\frac{N}{2})\theta }],$$

and the spatial profile $f(r)={\cosh }^{-1}[(r-R)/\xi ]$ closely resembles the vortex donut profile. We have chosen the width of the donut beam to be *ξ* = 0.2*R*.

**Figure 3:**
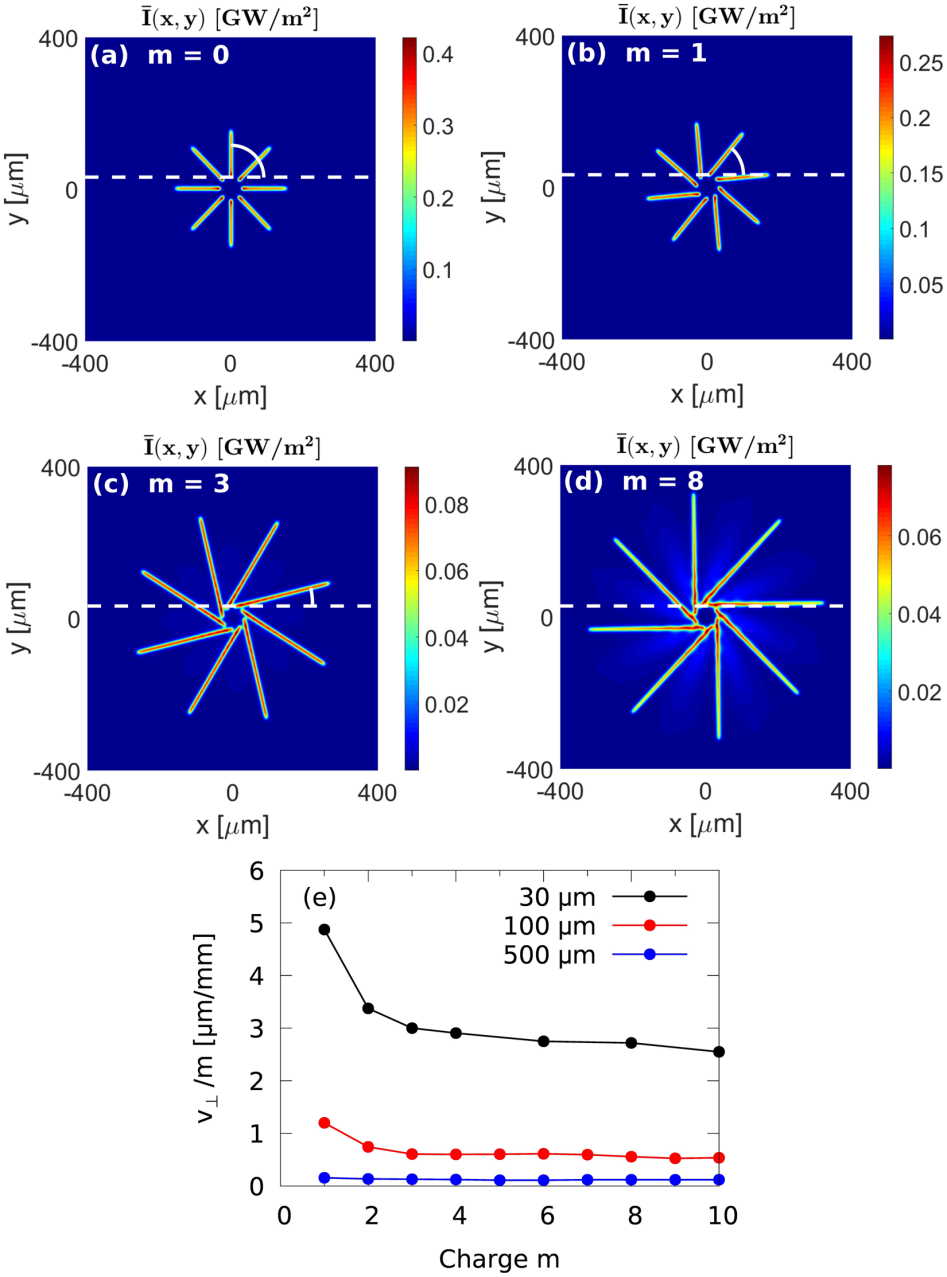
(**a**)–(**d**) Intensity maps averaged over the propagation distance [$\bar{I}(x,y)=\mathrm{1/}{z}_{{\rm{\max }}}{\int }_{0}^{{z}_{{\rm{\max }}}}I(x,y,z)\,{\rm{d}}z$] (${z}_{{\rm{\max }}}=40$ mm) for NBs with radius *R* = 30 *μ*m, eight maxima (solitons), input power 20 W, carrying various orbital angular momenta: *m* = 0 (**a**), *m* = 1 (**b**), *m* = 3 (**c**), and *m* = 0 (${z}_{{\rm{m}}{\rm{a}}{\rm{x}}}=20$ mm) (**d**). The white dashed line is tangent to the NB ring at the point located at the uppermost maximum for *z* = 0. (**e**) Transverse escape velocity of the solitons normalized by the beam OAM (${v}_{\perp }/m$) for various initial vortex radii: 30 *μ*m (black), 100 *μ*m (red), and 500 *μ*m (blue).

Figure 3(a)–(d) show the *z*-averaged intensity maps, $\bar{I}(x,y)=\mathrm{1/}{z}_{{\rm{\max }}}{\int }_{0}^{{z}_{{\rm{\max }}}}I(x,y,z)\,{\rm{d}}z$, that illustrate the trajectories of the soliton beams during the escape from the initial NB ring with the radius *R* = 30 *μ*m. The angle between the trajectory of the uppermost soliton and the line tangent to the NB ring at *z* = 0 is shown in the figures. We find that for charge *m* = 0, the solitons propagate in the direction perpendicular to the NB ring (right angle between the beam trajectory and the tangent line). This movement is caused by the diffraction of the NB. As the charge of the NB increases, the angle between the beam trajectory and the tangent line decreases. The velocities of the solitons have now two components: the component perpendicular to the tangent line, stemming from the beam diffraction; and the component parallel to this line, originating from the OAM carried by the NB. For high charges of the beam [e.g., *m* = 8 shown in Fig. 3(d)], the contribution of diffraction is negligible compared to the OAM-induced velocity, and the beams travel along the trajectories quasi-tangent to the NB ring. We have performed similar studies for NBs with radii *R* = 100 *μ*m and *R* = 500 *μ*m, and the results are summarized in Fig. 3(e). As expected, for beams with larger radius, the contribution of diffraction is smaller and it is visible only for low charges *m* of the beam. For radius *R* = 500 *μ*m the contribution of diffraction is negligible for the whole range of charges studied and the theoretically predicted relation ${v}_{\perp }/|m|={\rm{const}}(R)$ is fulfilled.

In the previous paragraphs, we have studied situations in which the solitons building the NB were traveling away from the vortex ring during their propagation. Different scenarios are also possible, in which the solitons forming the NB interact with each other. For the case of two interacting solitons (*N* = 2) spiraling and elliptical rotating beams can be formed. Spiraling solitons are build of two separate beams rotating around each other as they propagate. Generation of stable spiraling solitons was theoretically and experimentally demonstrated in materials with nonlocal nonlinearities^8,9,11–13,15–19^. Since the nonlinearity in our model is assumed to be local and instantaneous, the spiraling beams are unstable in such a medium^54^. Above the critical radius, the two beams escape from each other; and below this radius, the beams fuse. This latter phenomenon generates elliptical rotating beams: corkscrew-like beams where two tightly spaced solitons rotate around the common center of mass.

**Figure 4:**
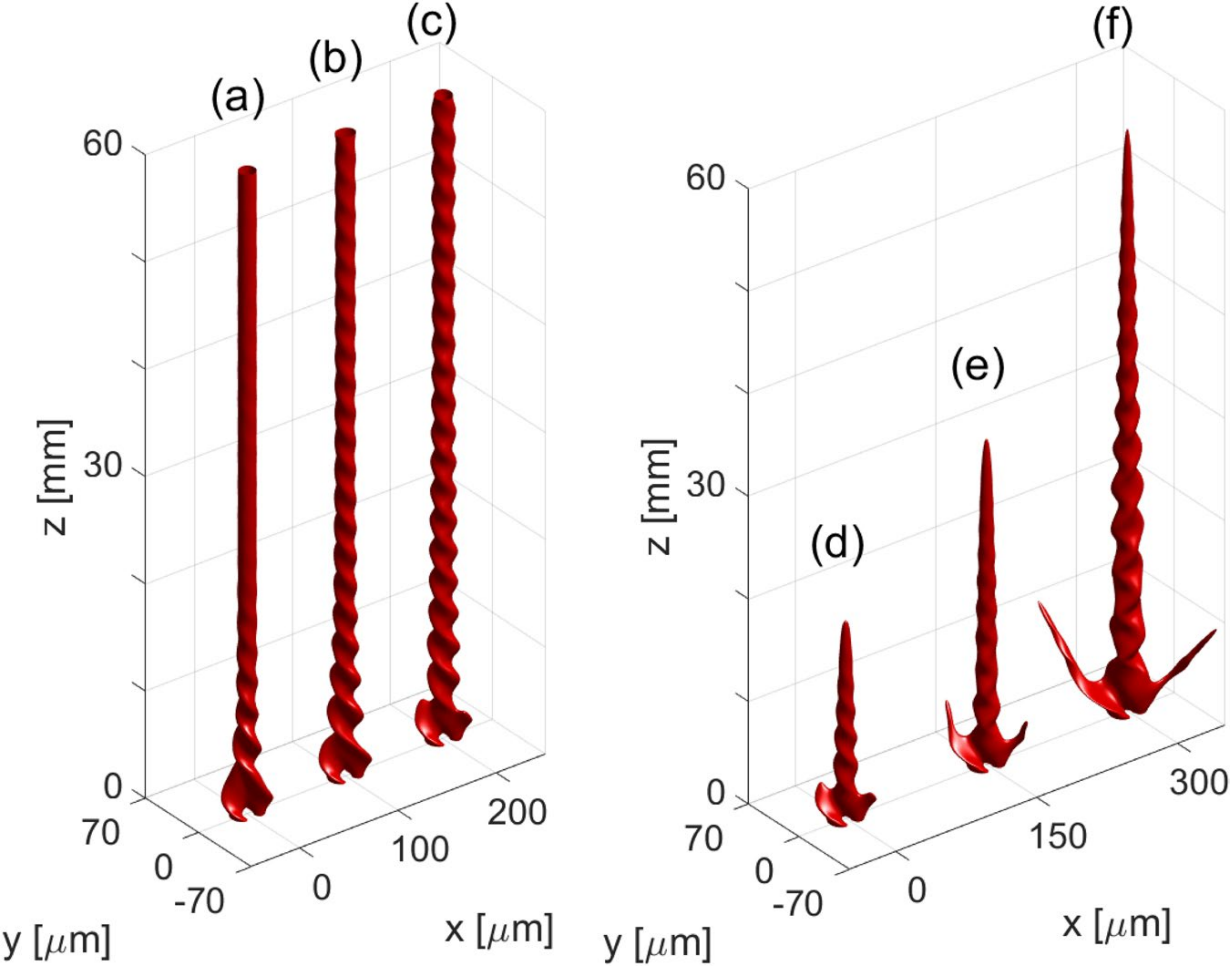
Elliptical rotating solitons generated in systems without loss (**a**)–(**c**) and with loss (**d**)–(**f**). The input is a NB with *m* = 1, *R* = 10 *μ*m, and two maxima. The input power levels are: 4 W (**a**), 6 W (**b**), 8 W (**c**), 12 W (**d**), 20 W (**e**), and 40 W (**f**).

Figure 4 shows the propagation of elliptical rotating beams in lossless and lossy media for various input-power levels. In the lossless case, at low power, the elliptical rotating beam is formed at the beginning of the propagation, but after approximately 20 mm, the rotation is no longer visible and the beam propagates as a regular soliton. The excess of energy, which is a result of the fusion of the two beams, is expelled away from the main fundamental soliton beam and only a single stable soliton remains. For higher input energies, the twisted nature of the beam is preserved for a longer propagation distance, as it takes more time to expel the energy excess. Increase of the energy does not allow for formation of higher-order solitons because the required nonlinear index modification exceeds the maximum attainable index change in the saturable CS studied here. In the case of propagation in the lossy medium, the elliptical rotating beam maintains the twisted character and propagates for longer distance as the power is increased, due to the self-induced transparency effect in the negative polarizability CSs^43^.

**Figure 5:**
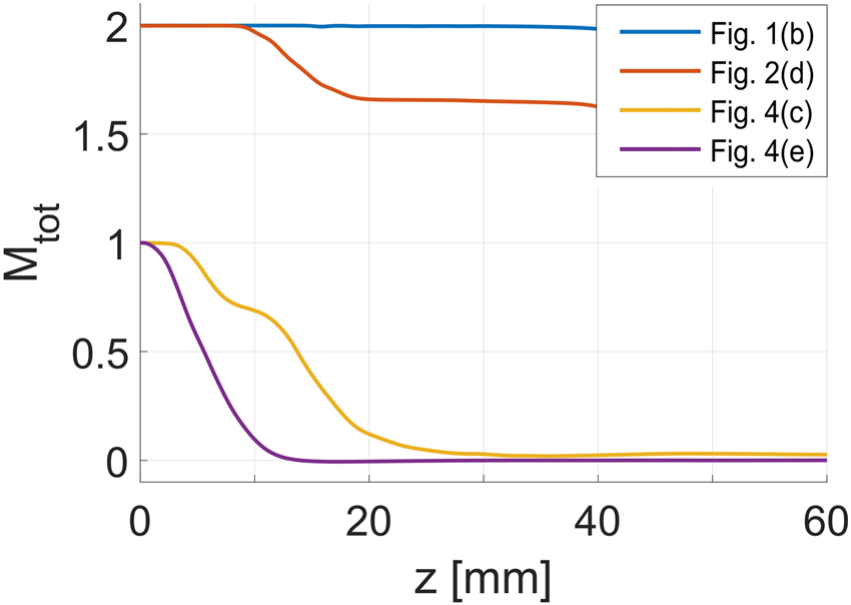
The total OAM as a function of propagation distance for necklace beams (blue and red curves) and elliptical rotating beams (orange and violet curves) shown in the plots indicated in the legend. OAM conservation during propagation in colloidal media without loss (blue and orange curves), and with loss (red and violet curves) is analyzed.

Finally, we have studied the conservation of the OAM of the NBs and the elliptical rotating beams. The total OAM is calculated as:

3 $${M}_{{\rm{tot}}}=\frac{\frac{i}{2}\iint {\bf{r}}\times (\varphi {\nabla }_{\perp }{\varphi }^{\ast }-{\varphi }^{\ast }{\nabla }_{\perp }\varphi )\,{\rm{d}}x{\rm{d}}y}{\iint |\varphi {|}^{2}\,{\rm{d}}x{\rm{d}}y},$$

where **r** = [*x*, *y*] is a vector of position in the transverse plane and ${\nabla }_{\perp }=[\frac{\partial }{\partial x},\frac{\partial }{\partial y}]$. Figure 5 presents plots of the total OAM as a function of the propagation distance for four beams studied above: two NBs and two elliptical rotating beams. Blue and red curves show ${M}_{{\rm{tot}}}(z)$ for NBs without and with losses, respectively. In the lossless case, for which the iso-intensity surface is shown in Fig. 1(b), the OAM is conserved both when the vortex beam propagates and transforms into the NB, and during the propagation of the generated solitons. In the case of the NB generation in a lossy medium [shown in Fig. 2(d)], the OAM is not fully conserved. It is conserved during the propagation of the vortex beam, but when the NB is formed, part of the OAM is lost. After the solitons lose energy and diffract, the OAM does not decrease any more. The loss of the OAM in the middle stage of the propagation is caused by fact that the three solitons forming the NB do not have equal energies, as they grow from random noise due to the MI. In our model, loss is intensity dependent, and the solitons with the lower power lose the energy faster than the solitons with higher power. This results in a non-symmetric distribution of the energy and leads to changes in the OAM carried by the NB. When beam intensities decrease, all the beams decay at the same rate (corresponding to the loss level in the linear regime), and the OAM of the linearly diffracting field becomes conserved again.

For the elliptical rotating beams resulting from the fusion of two solitons, the OAM is not conserved, as shown by the orange and violet curves Fig. 5. Here, the OAM is expelled from the elliptical rotating high-intensity beam and it is carried by the low-intensity field (not visible in iso-intensity plots) away from the central beam. The confirmation of this hypothesis can be seen comparing the plots of the OAM and the power evolution, shown in the videos provided in the Supplementary Materials. During the lossless propagation shown in Fig. 4(c), the low-intensity field carrying the OAM is absorbed when it reaches the simulation domain boundaries. Surprisingly, the twisted character of the beam is present, despite the fact that the beam does no longer carry the OAM. In the system with losses [see Fig. 4(e) and the corresponding violet curve in Fig. 5], the low-intensity field that carries the OAM is rapidly scattered by the CS. As a result, the OAM in this system decreases to zero in the first 10 mm of propagation.

In conclusion, we have investigated the dynamics of vortices in nonlinear colloidal suspensions and showed that the distance at which the modulation instability transforms a vortex beam into a necklace beam depends on the input power, and that the minimum distance is achieved for the power corresponding to the stationary vortex soliton. Moreover, we have demonstrated the influence of diffraction on the trajectories of necklace beams and the value of the escape velocity. The solitons forming the necklace beam have been shown to follow linear trajectories that are not necessarily tangent to the necklace beam ring, and the angle of escape can be controlled by the interplay of the necklace beam radius and its charge. We have presented the possibility of elliptical rotating soliton formation using necklace beams with two maxima. Finally, we have analyzed the influence of losses and the conservation of orbital angular momentum in the necklace beams and elliptical rotating beams. These results may be of interest for the formation of complex photonic structures in liquids as well as in the broader field of light filamentation.

### Data Availability

No datasets were generated or analyzed during the current study.

## Electronic supplementary material

Supplementary informations

Fig1a

Fig1bLARGE

Fig1b

Fig1c

Fig2a

Fig2b

Fig2c

Fig2d

Fig3a

Fig3b

Fig3c

Fig3dLARGE

Fig3d

Fig4a

Fig4b

Fig4c

Fig4d

Fig4e

Fig4f
