# Supplementary material for: Dynamics of necklace beams in nonlinear colloidal suspensions
Source: Sci Rep. 2017 Sep 15;7:11709. doi: 10.1038/s41598-017-12169-x (PMC5601962; doi:10.1038/s41598-017-12169-x)
Supplement: Supplementary file 1 — Supplementary informations [file 41598_2017_12169_MOESM1_ESM.pdf]

Supplementary material for
"Dynamics of necklace beams in nonlinear colloidal suspensions"

Wiktor Walasik,[1] Salih Z. Silahli,[1] and Natalia M. Litchinitser[1]

[1] *Department of Electrical Engineering, University at Buffalo, The State University of New York, Buffalo, New York 14260, USA**

I. MODULATION INSTABILITY ONSET IN MEDIUM WITH SATURABLE NONLINEARITY

The results presented in Fig. 1 in the main text of the manuscript show that, above the power level corresponding to the stable vortex solution, the modulation instability (MI) onsets at a longer propagation distance than the MI-onset distance for the stable vortex. Figure S1 provides a phenomenological explanation of this behavior. Here, we consider a nonlinear colloidal medium with a saturable nonlinearity and the parameters specified in the main text of the manuscript. According to the model described by Eq. (1) in the main text of the manuscript, the nonlinear refractive index change in this medium is given by $\Delta n = (n_p - n_b)V_p\rho_0\left[1 - e^{\frac{\alpha}{4k_BT}|\phi|^2}\right]$, where $\alpha < 0$. Figure S1 shows the normalized nonlinear refractive index change $\delta n = \Delta n/\Delta n_{\max}$ as a function of light intensity, where $\Delta n_{\max} = (n_p - n_b)V_p\rho_0$ denotes the maximum possible value of the nonlinear index change reached in the saturation regime. The light intensity I is related to the field amplitude ϕ as $I = n_b\epsilon_0 c/2|\phi|^2$, where ϵ_0 denotes the vacuum permittivity and c is the speed of light in vacuum.

Let us consider two beams with different peak intensities: the low intensity, below the saturation regime; and the high intensity, close to the saturation regime. For both beams, we assume a high noise with the relative amplitude of 20% of the peak intensity, in order to clearly illustrate the differences of the MI behavior in these two regimes. From Fig. S1, we observe that, even though the absolute amplitude of the intensity variations is larger for the high-intensity beam, the resulting nonlinear refractive index change $\delta n_{\rm sat}$ is much smaller than the index change in the low-intensity regime $\delta n_{\rm low}$.

The MI occurs as a consequence of the amplification of the noise present in the initial beam profile [1]. The beam profile without noise leads to a smooth and regular refractive index distribution. On top of this regular index profile, the noise induces refractive index irregularities that result in self-focusing of the light in the regions with higher refractive index. This leads to growth of perturbations with specific wave-vectors determined by the characteristics of the initial beam [2–6]. The growth rate of the perturbation is proportional to the amplitude of the irregularities in the refractive index distribution. As a result, in the saturation regime, the MI onsets at a longer propagation distance than for lower light intensities, below the saturation regime.

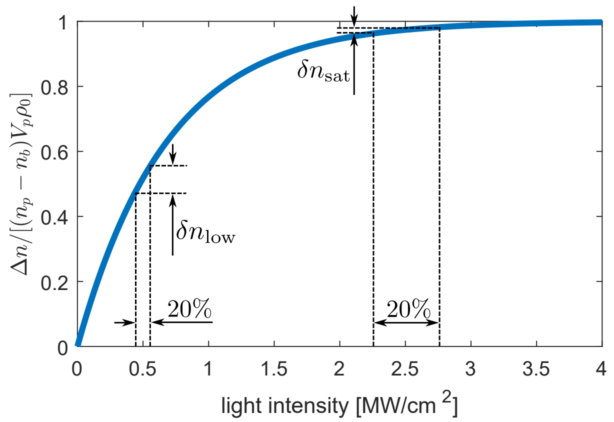

FIG. S1. Relative refractive index modification $\delta n = \Delta n/[(n_p - n_b)V_p\rho_0]$ as a function of the light intensity in the medium with saturable nonlinearity and with the parameters specified in the main text of the manuscript.

* wiktorwa@buffalo.edu

II. DESCRIPTION OF THE BEAM PROPAGATION VIDEOS

For each of the beams studied in this paper, we provide a video illustrating the evolution of the beam during the propagation. The simulations were performed using an in-house Split-Step Fourier solver. The simulation window was surrounded by a lossy region in order to prevent reflections from the boundaries of the simulation window. The losses were mimicked by attenuating the electric field close to the simulation domain boundaries using a super-Gaussian function $L(x, y) = \exp\{-[x/(0.45D)]^{30}\}\exp\{-[y/(0.45D)]^{30}\}$, where D is the total size of the simulation window. At every step of the simulation the electric field was multiplied by $L(x, y)$.

In each video, we present five panels showing the evolution of:

1. Modified light intensity profiles in the transverse plane $I_m(x, y)$ at different propagation distances z.

   The modified light intensity profile is defined as $I_m(x, y) = I(x, y)+\max\{I(x, y)\}\cdot[1-L(x, y)^{-1}]$. This definition allows us to convey two types of information in one color-map:

   - in the center of the simulation domain [where $L(x, y) \approx 1$ and losses imposed by a super-Gaussian function are absent] the color-maps preset the real light intensity profiles $I(x, y)$,
   - the location of the lossy boundaries that are represented by a high intensity region close to the simulation window borders.

2. Local orbital angular momentum distribution in the transverse plane,

$$M_{\mathrm{loc}}(x, y) = \frac{\frac{i}{2}\mathbf{r}\times(\phi\nabla_\perp\phi^* - \phi^*\nabla_\perp\phi)}{|\phi|^2}, \tag{S.-2}$$

   at different propagation distances z.

3. Phase of the electric field ϕ in the transverse plane at different propagation distances z.

   In the color-maps showing the local orbital angular momentum (see point 2) and the phase of the electric field, these quantities are shown only in the regions with the local light intensity higher than a certain threshold intensity. The intensity threshold was chosen to be $I_{\mathrm{th}}(z_0) = \max\{I(x, y, z = z_0)\}/100$.

4. Evolution of the total orbital angular momentum $M_{\mathrm{tot}}(z)$ as a function of the propagation distance.

5. Evolution of the total power of the beam $P_{\mathrm{tot}}(z) = \iint I(x, y, z)\,\mathrm{d}x\mathrm{d}y$ as a function of the propagation distance.

The hypothesis proposed in the main text of the manuscript concerning the OAM conservation in elliptical rotating beams is supported by the plots of $M_{\mathrm{tot}}(z)$ and $P_{\mathrm{tot}}(z)$. Looking at these plots, we can see that the total OAM decreases at the same propagation distances as the total power. The curvatures of both plots are very similar which suggests that the two phenomena (decrease of the OAM and decay of the power related due to losses) are closely related.

[1] R. W. Boyd, ed., "Nonlinear optics," (Academic, New York, 2007) Chap. Processes Resulting from the Intensity-Dependent Refractive Index, pp. 329–390.
[2] W. J. Firth and D. V. Skryabin, Phys. Rev. Lett. **79**, 2450 (1997).
[3] D. V. Skryabin and W. J. Firth, Phys. Rev. E **58**, 3916 (1998).
[4] A. Vincotte and L. Berg, Physica D **223**, 163 (2006).
[5] L. T. Vuong, T. D. Grow, A. Ishaaya, A. L. Gaeta, G. W. 't Hooft, E. R. Eliel, and G. Fibich, Phys. Rev. Lett. **96**, 133901 (2006).
[6] S. Z. Silahli, W. Walasik, and N. M. Litchinitser, Opt. Lett. **40**, 5714 (2015).
